# Quantum Key Based Burst Confidentiality in Optical Burst Switched Networks

**DOI:** 10.1155/2014/786493

**Published:** 2014-01-22

**Authors:** A. M. Balamurugan, A. Sivasubramanian

**Affiliations:** Department of Electronics and Communication Engineering, St. Joseph's College of Engineering, Chennai 600119, Tamil Nadu, India

## Abstract

The optical burst switching (OBS) is an emergent result to the technology concern that could achieve a feasible network in future. They are endowed with the ability to meet the bandwidth requirement of those applications that require intensive bandwidth. There are more domains opening up in the OBS that evidently shows their advantages and their capability to face the future network traffic. However, the concept of OBS is still far from perfection facing issues in case of security threat. The transfer of optical switching paradigm to optical burst switching faces serious downfall in the fields of burst aggregation, routing, authentication, dispute resolution, and quality of service (QoS). This paper deals with employing RC4 (stream cipher) to encrypt and decrypt bursts thereby ensuring the confidentiality of the burst. Although the use of AES algorithm has already been proposed for the same issue, by contrasting the two algorithms under the parameters of burst encryption and decryption time, end-to-end delay, it was found that RC4 provided better results. This paper looks to provide a better solution for the confidentiality of the burst in OBS networks.

## 1. Introduction

The beginning of new millennium provides several new trends in the field of communication network. During the decades there is always a demand for transmission bandwidth and this demand grew without limit. The movement of traffic from one part of the network to another part is provided by the switches. Generally, the capacity of a switch is to maximize the rate such that the switch can move the information assuming all data parts are sent. They are concerned in moving the data from one node to the other node until the respective destination is reached. On the other hand the switched network cannot transmit bulk data on the entire medium. Hence data transmission from source to destination is carried out by the intermediate nodes [1].

The basic switching concept involves circuit switching and packet switching. For an optical transport network, their advantages and disadvantages are characterized. During the early generations, circuit switching was carried out by using copper wires through a number of electronic circuits. As the demand for bandwidth increases in the network, these copper wires were replaced by optical fiber [2]. These optical fibers can carry a large amount of bandwidth. Further the bandwidth of the optical fiber can be exploited by the use of WDM technology. The main objective of this switching method is to assign specific wavelength to each source to destination pair. Optical circuit switching (OCS) handles a huge amount of long lived data transfer. But before the transmission of the data packets, a well-established data path is required. This causes the reservation of channel capacity between the respective sources to destination pair throughout the network [3]. That is, each source to destination pair requires a specific wavelength (*λ*), where a light path should be dedicated to the respective source to destination pair. This technique is not scalable, since when *N* nodes are used, the requirement of wavelength will be *O*(*N* ^2^) which is practically impossible because of virtual point-to-point link. They require a certain amount of time for establishing a channel independent of the connectivity holding time. Moreover in optical circuit switching the adaptation to the traffic matrix is not possible [3]. This circuit switching concept has been replaced by a competitor such as packet switching. This is an alternate paradigm to overcome the pitfalls in optical circuit switching. Packet switching governs how messages should be transmitted between two points. They divide a long message into pieces called packets. Each packet contains a header. Packet switching is connectionless. They require routers and routing algorithms. The advantage of packet switching is that they do not need resources all the time. Transmission resources are available only when the data is available [4].

In optical packet switching (OPS) at each intermediate node the packet header is processed either optically or electronically after O/E conversion. This processing can be classified into two major fields. They are transparent OPS and all OPS. Transparent OPS defines the processing of packet header electronically. Whereas all OPS deals with the processing of packet headers optically. Until the conversion process is completed the corresponding data payload waits in the fiber line and then they are forwarded to the next node. In the switching concept the wavelength capacity is dynamically time shared with a limited number of available wavelengths [5]. Here the links are occupied depending on the demand. In case of congestion occurrence an alternate route will be provided [6].

Though this method is more advantageous than optical circuit switching, they too exhibit certain disadvantages. There is a lack of optical random access memory. Since they are connectionless, adaptation to ultra-switching requirement is not possible. The packets are limited due to various reasons that arise from the stem factor. As each node requires buffering capacity, there will be a delay if forward and store is used. Here the links are occupied depending on the demand. These methods provide certain description for the internet traffic density. Due to the technology development and the user request, the need for bandwidth is huge. This fact has triggered the elimination of optical packet switching through electronic processing of headers for routing process.

**Figure 1:**
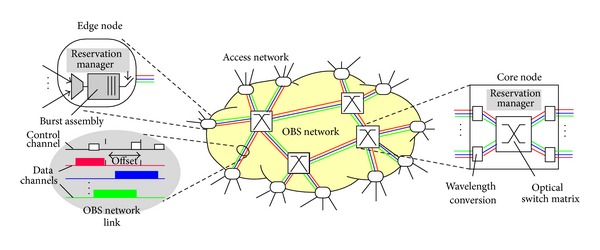
OBS architecture [7]. Courtesy: Gauger et al. [7].

The introduction of a new paradigm which combines both the advantages of optical circuit switching and optical packet switching is the optical burst switching. OBS architecture consists of an edge node and a core node is shown in Figure 1. Edge node consists of ingress node and egress node. Ingress node assembles the data packets from various sources into a burst. Bursts may have variable sizes. Egress node disassembles the burst into packets again [7].

## 2. End-to-End Data Burst Confidentiality

In OBS networks, the ingress edge router consists of the collection of data which stay in the OBS core network in the optical domain and only at egress edge router they are disassembled. By encrypting data bursts at the ingress edge router and decrypting at the egress edge router the data bursts switch transparently across the OBS core routers, so the end-to-end burst confidentiality within the OBS domain is provided [8]. In this OBS technique, when a burst is passed from ingress node to egress node through many intermediate core nodes, it can be possibly stolen by the attacker nodes [9]. How the attacker nodes use the various methods to steal the burst and how we can prevent this from happening are discussed below.

Physically tapping in an optical fiber is one of the methods. Residual crosstalk from an adjacent channel can be heard while impersonating a legitimate subscriber which is another method used by the attacker. In the first method mentioned, if a fiber is exposed with no physical protection, then tapping an optical fiber can be done by peeling off the cladding of the fiber and the protective material enhancing the escape of a small part of the light from the optical fiber. Also, by placing another fiber adjacent to the path of escape of the light, a small portion of the desired optical signal can be captured. But, practically, tapping in this way is not that easy as only a marginal amount of signal tapping can be done and the excessive power lost in the optical signal remains unnoticed. Another practical difficulty in this method of tapping is the peeling off cladding and protective material from the fiber as it can quite easily lead to breakage, the reason being that almost all the optical fibers available in communication systems are usually bundled and consist of cabling and protective materials in a multiple-layer form thereby concluding that tapping an optical fiber physically is a difficult task. The second method can be possibly used in WDM (wavelength-division-multiplexing) networks where various subscribers use various wavelengths and where a wavelength demultiplexer is used to drop a desirable signal at its destination. But there is an imperfection in channel isolation in the wavelength demultiplexers thereby showing a minimal leakage of optical power from the adjacent channels which is called interchannel crosstalk [10]. Hence, the residue signal can be obtained by the eavesdropper by listening to the leakage from the adjacent channel. The two methods discussed above though are not practically easy to bring into implementation still show possibility when eavesdroppers are available with specialized optical equipment. To protect the optical network or improvise on the confidentiality aspect, two methodologies, namely, optical encryption and optical coding, can be implemented. In a physical layer, securing a signal and hence improvising on confidentiality of the network are possible through encryption. Data recovery from the cipher text is impossible unless the eavesdropper has the knowledge of the encryption key. The advantage here is that even if the eavesdropper is successful in obtaining a portion of signal by tapping, no appropriate information can be got without knowing the encryption key.

The existing approach uses the advanced encryption standard (AES) for data bursts securing. The proposed approach uses stream cipher based RC4 algorithm for data burst confidentiality. Moreover we use quantum based key distribution schemes for transferring the keys.

The rest of the paper is organized as follows.  Section 3 gives the overview of quantum key generation for our burst encryption.  Section 4 will have our proposed burst encryption scheme. Finally we discuss our results in Section 5.

## 3. QKD Based Key Generation for Burst Encryption

Cryptography systems can be classified into symmetric-key systems and public-key systems. Symmetric-key cryptography is an encryption technique in which both the sender and receiver share the same key. Symmetric-key cryptosystems use the same key for encryption and decryption of a message, while a message or group of messages may have a different key than others. A drawback of symmetric ciphers is the key management required to use them securely. Here we are generating a symmetric key using quantum key distribution for encrypting and decrypting the burst.

It is proven that when the length of the message (in other words, if the rate at which the key can be transported) equals the data speed, the encryption performed on the message through a simple technique such as exclusive OR operation will be theoretically unbreakable cipher [11]. Since there is no secure way of sending the random key over a public channel, the use of quantum cryptography can be envisaged as matching the performance of the theoretically unbreakable cipher. In short, quantum cryptography is ideally suited for OBS since it is fundamentally based on the quantum properties of the photons. Besides leading to a theoretically unbreakable scheme, the quantum based encryption technology is well matched for use in an end-to-end photonic environment, which the OBS environment typifies.

### 3.1. Quantum Cryptography

It is built based on Heisenberg's uncertainty principle. It states that certain pairs of physical properties cannot be calculated simultaneously [12]. If one of them is calculated, the other gets disturbed and becomes impossible to compute. Quantum key distribution uses a separate channel to transmit key. It carries photons of random polarization and is known as Q-Bits. Photons get altered when they are measured. Thus data through this channel cannot be intercepted without being detected. This is achieved by sender encoding the bits of the key as quantum data and sending them to receiver. If third party tries to learn these bits, then the messages will be disturbed and both the sender and receiver will notice thereby making it unbreakable. The key is thus typically used for encrypted communication. The security of QKD can be proven mathematically without imposing any restrictions on the abilities of an eavesdropper, something that is not possible with classical key distribution. This is frequently described as “unconditional security” even though there are some minimal assumptions required including that the laws of quantum mechanics apply and that Sender and Receiver are able to authenticate each other, that is, third party should not be able to impersonate Sender or Receiver as otherwise a man-in-the-middle attack would be possible. QKD is the only example of commercially available quantum cryptography. There are three main security protocols for QKD, namely, B92, BB84, and entanglement based QKD. In this paper we use two-stage quantum key generation using B92 and BB84 protocols. In BB84 protocol we use two polarization states called rectilinear (R) and diagonal (D). The single photon could be polarized as four states: H, V, |45°〉, and |135°〉 which are shown in Table 1.

### 3.2. B92 Protocol Based Key Generation

B92 uses only two states at ingress node and two states at egress node. B92 protocol begins with the ingress node sending a random sequence of photons, H-*photon* and 135°-*photon*. Egress node randomly chooses one of its detector basis, 45°-*basis*, or V*-basis* and records its measurement results (yes or no). Egress node sends a copy of its results to ingress through the public channel. Finally, ingress and egress will keep the bits where the results are “Y,” neglecting all other bits which are shown in Table 2.

The basic procedure involved can consolidate as follows. Ingress node sends a random sequence of photons, H-*photon* and |135°〉-*diagonal photon*. Then, Egress node randomly chooses its detector basis from |45°〉-diagonal *basis* or V*-basis* to measure each photon, and the bases are interpreted as a binary sequence. Results of egress node's measurement are taken. Ingress and egress will share the bits where the measurement results are “Y,” discarding all other bits. Thus the efficiency of this protocol is 25%. For a *k* sequence in the B92 protocol, the idealized maximum shared bits between ingress and egress nodes are *k* × 1/4. Thus the complexity order for this protocol can be calculated as follows. There are *k* photons with two polarization states. The average complexity order is (2 × *k*)/*k* = 2.

### 3.3. BB84 Protocol Based Key Generation

This protocol is proposed by Bennett and Brassard in 1984. This is known to be the first quantum cryptography protocol. It is a quantum primitive that can be used in different kinds of protocols.

Ingress node sends a random sequence of photons, |H〉-*photon*, |V〉-*photon*, |45°〉-*photon*, and |135°〉-*photon*. Egress randomly chooses its detector basis from R-*basis* or D-*basis* to measure each photon. Results of egress node's measurement are taken. Then, the states are interpreted as a binary sequence. Egress node reports its detector bases for each photon. Ingress tells egress which bases were correct. Finally, ingress and egress will share the bits where ingress node's response is “Y,” discarding all other bits which are shown in Table 3. Efficiency is 25% for B92 and 50% for BB84. But the complexity order is increased to 4.

### 3.4. Two-Stage QKD Protocol (Stage 1: B92 and Stage 2: BB84)

This protocol makes use of both B92 and BB84. In the first stage, ingress node sends a random sequence of photon using B92, and in the second stage, egress node will use BB84 or B92 to send the photons in which egress node's measurement results are “N” in the first stage.

Ingress node sends a random sequence of photons, |H〉-*photon *and |135°〉-*diagonal photon*. egress node randomly chooses its detector basis from |45°〉-diagonal *basis* or |V〉*-basis *to measure each photon, and the bases are interpreted as a binary sequence. Results of Egress node's measurement are taken. Egress node chooses its basis according to egress node's bit, and then it sends a random sequence of photons, |H〉-*photon*, |V〉-*photon*, |45°〉-*photon*, and |135°〉-*photon* where its measurement results are “N”. Ingress chooses its detector bases according to ingress node's bits. To measure each photon, results of ingress node's measurement are taken. Then, the states are interpreted as a binary sequence. Ingress node reports its detector bases for each photon. Egress tells ingress which bases were correct. Ingress and egress will share the bits where the results are “Y” in the 2nd stage, neglecting all other bits. Ingress and egress will get the final shared secret Key which are shown in Table 4.

For a *k*-bit sequence in the two-stage QKD protocol, the idealized maximum shared bits between ingress node and egress node are *k* × 1/4 in the first stage. Egress node resends 3*k*/4 bits in the second stage and ingress node may get maximum correct 3*k*/4 × 2/30 bits. Finally the efficiency of our two-stage QKD protocol will be (*k* × (1/4)+(3*k*/4)×(2/3))/(*k* + (3*k*/4)) = 42.9%.

Thus, the average complexity order of two-stage QKD is calculated as follows. There are *k* photons with two polarization states in the 1st stage (B92) and 3*k*/4 photons with four polarization states in the 2nd stage (BB84). The average complexity order is given by ((2 × *k* + 4 × ((3 × *k*)/4)))/((*k* + ((3 × *k*)/4))) = 2.86. The complexity order and efficiency of various QKD protocols are shown in Table 5.

## 4. Burst Encryption Algorithm

In optical burst switching for providing more security cryptography technology is used which is termed as cipher. Moreover ciphers can be distinguished into two types by the type of input data; they are *block ciphers*, which encrypt block of data of fixed size, and *stream ciphers*, which encrypt continuous streams of data. The block ciphers use the AES algorithm and the stream cipher uses the RC4 algorithm [13].

### 4.1. Block Cipher

*Block cipher* is an algorithm operating on blocks which is fixed length of bits; the blocks which have the fixed length of bits are encrypted using the symmetric key [14]. Block ciphers are broadly used to implement bulk data encryption and it is the basic components in many cryptographic protocols. In block cipher for encryption it allows single data block of its cipher's block length. In this for the cipher's block size the message is divided into separate blocks and then the encryption and decryption for each block are done independently. If we use the block cipher in our optical burst switching for the bursts, the block cipher will produce the same key so it will become easy to hack the key, so the security in burst transferring is at high danger. So, in this method the security is very poor because using the same key for the plaintext blocks which is the input generates equal output ciphertext blocks. AES algorithm is the most preferred algorithm which is used in the block cipher.

A substitution permutation network is the principle of the AES algorithm. In this algorithm for both encryption and decryption the same key is used. The AES algorithm key sizes can be of 128, 192, or 256 bits. For 128 bit keys the number of cycles of repetition is 10 cycles, for 192 bits the number of cycles of repetition is 12 cycles, and for 256 bit keys the number of cycles of the repetition is 14 cycles.

There are many disadvantages of block cipher such as it is easy to insert or delete blocks. In block cipher identical block of plain text yields identical blocks of cipher text so it is easy to modify by the common user. To overcome all these disadvantages we are using stream cipher to encrypt the burst instead of block cipher.

### 4.2. Stream Cipher

Stream cipher is of a symmetric key cipher and each plaintext digit is encrypted one at a time to produce the cipher output. An alternate name for the stream cipher is state cipher, as each digit data encryption is dependent on the current state only. As in optical burst switching by encrypting each digit the security is added and the time for processing is minimized. Stream ciphers have lower hardware complexity and execute at a higher speed than block ciphers. Stream ciphers are also best for cases where the amount of data is either unknown or continuous—such as network streams [15].

Stream ciphers are really suitable for hardware implementation that uses one bit data at a time for the encryption and the decryption. Stream cipher is less vulnerable to insertion or deletion of block. It can be mathematically analyzed easily. The key in the stream cipher is generated independently of the message stream. Thus stream cipher is well suited for burst switching compared to the block cipher.

In the optical burst switching, stream cipher is used and the better algorithm used for the cipher is RC4, and the symmetric key for the stream cipher is generated using the quantum key distribution (QKD). The already recommended algorithm is AES which has some sort of disabilities which have been discussed above so, to overcome the disadvantages of using AES, the RC4 algorithm is recommended for the secure burst switching and the key generation for the ciphering is done with Quantum Key Distribution (QKD).

RC4 is a stream cipher designed in 1987 by Ron Rivest. It is officially termed as “Rivest Cipher 4.” Stream ciphers are more efficient for real time processing. It is a variable key size stream cipher with byte oriented operations. This algorithm is based on the use of a random permutation. Eight to sixteen machine operations are required per output byte and the cipher runs very quickly in software. It can be efficiently implemented in both software and hardware.

### 4.3. Advantages of Using RC4

In the optical burst switching for providing the security the stream cipher is used which works on the RC4 algorithm. In cryptography, RC4 is also known as ARC4 meaning alleged RC4 and used most widely in cipher for its remarkable simplicity and speed of processing [16]. It uses the pseudorandom generation algorithm so it provides more security. The key generation is using quantum key distribution.

The next section will give the performance comparison of block cipher and stream cipher based burst encryption and decryption.

## 5. Results and Discussions

**Figure 2:**
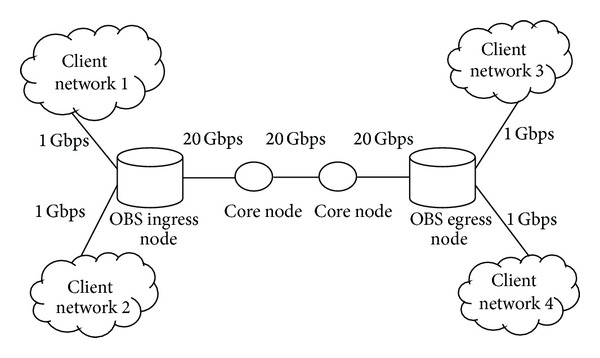
Simulation scenario.

Our simulation scenario is depicted in Figure 2. For our simulation we consider two client networks connected to an OBS ingress node via 1 Gbps link. The ingress nodes are connected via core nodes with the link capacity of 20 Gbps. The client network will generate the packets with the size of 1500 bytes/packet. The ingress node will aggregate these packets into burst. The burst size will vary from 40000 packets/burst to 1, 20,000 packets burst. We encrypt the burst using RC4 and AES algorithms with the hardware specification of 2.99 GHZ CPU and 2 GB RAM, in which data collected are shown in Tables 6 and 7.

**Figure 3:**
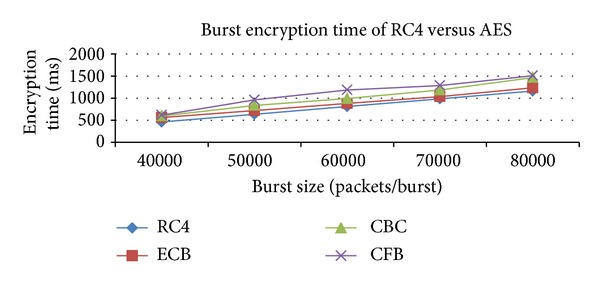
Comparison of burst encryption time with various algorithms.

**Figure 4:**
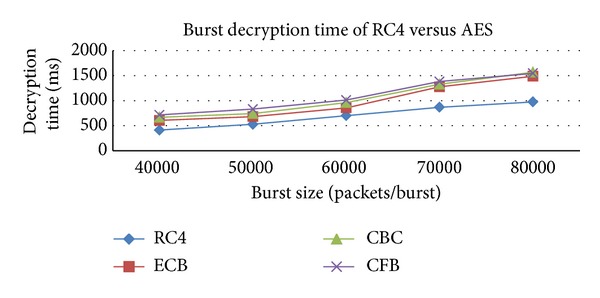
Comparison of burst decryption time with various algorithms.

From this simulation, we are trying to find out the performance comparison of encryption and decryption time between Block cipher and Stream cipher algorithms for burst encryption. These results show that the RC4 algorithm gives better result than AES algorithm for burst encryption and decryption are shown in Figures 3 and 4.

### 5.1. End-to-End Delay Calculation

**Figure 5:**
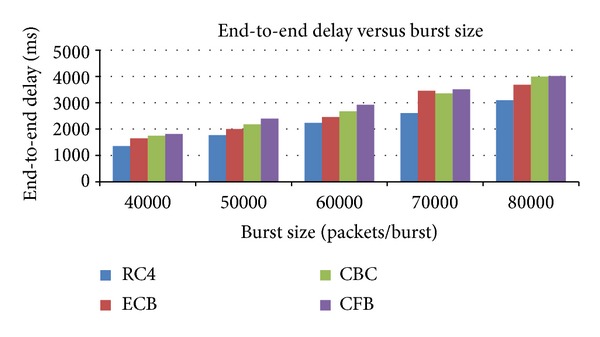
End-to-end delay of a burst using various encryption and decryption algorithms.

The end-to-end delay refers here to the time taken by the burst to travel from ingress node to egress node. This delay is the sum of burst assembly time, burst encryption and decryption time, and header processing time in an intermediate core routers which are shown in Table 8. Here we are taking our interest to reduce the delay for burst encryption and decryption which is shown in Figure 5. Obviously our results show our proposed stream cipher based RC4 will take lesser time to encrypt and decrypt our burst than the existing AES approach:

 end-to-end delay time = *T*_*b*_ + *T*_Eb_ + (*h* × *T*_offset_) + *T*_Db_
 
*T*_*b*_ = burst assembly period at edge node
 
*T*_Eb_ = burst encryption time
 
*h* = number of hops (core node)
 
*T*_offset_ = offset time of the control packet in the *n* core nodes
 
*T*_Db_ = burst decryption time at edge node.

## 6. Conclusion

Thus, from, the results obtained for several sizes of bursts for both AES algorithm and RC4 algorithm, it is evident that the latter is superior when it comes to burst confidentiality as it requires less encryption and decryption time, therefore less end-to-end delay in OBS networks.

## Figures and Tables

**Table 1:** Polarization states for QKD.

State/bit	0	1
Rectilinear	H	V
Diagonal	∣45°〉	∣135°〉

**Table 2:** An 8-bit sample for B92 protocol.

Sequence of bits		1	2	3	4	5	6	7	8
1	Ingress node bit	1	0	1	0	1	1	0	0
	Ingress node polarization	135°	H	135°	H	135°	135°	H	H
2	Egress node detector basis	45°	45°	V	V	45°	45°	45°	V
	Egress node bit	0	0	1	1	0	0	0	1
3	Egress node measurement	N	N	Y	N	N	N	Y	N
	Shared secret key	—	—	1	—	—	—	0	—

**Table 3:** An 8-bit sample of BB84 protocol.

Sequence of bits		1	2	3	4	5	6	7	8
1	Ingress node bit	1	0	1	0	1	1	0	0
	Ingress node source basis	D	R	R	R	D	R	D	D
	Ingress node polarization	135°	H	V	H	135°	V	45°	45°
2	Egress node detector basis	D	R	D	R	R	R	R	D
3	Egress node measurement	135°	H	45°	H	H	V	V	45°
	Egress node bit	1	0	0	0	0	1	1	0
4	Egress node report basis	D	R	D	R	R	R	R	D
5	Ingress node response	Y	Y	N	Y	N	Y	N	Y
6	Shared secret key	1	0	—	0	—	1	—	0

**Table 4:** An 8-bit sample for two-stage QKD protocol.

Sequence of bits		1	2	3	4	5	6	7	8	
1	Ingress node bit	1	0	1	0	1	1	0	0	STAGE I
	Ingress node polarization	135°	H	135°	H	135°	135°	H	H	
2	Egress node detector basis	45°	45°	V	V	45°	45°	45°	V	
	Egress node bit	0	0	1	1	0	0	0	1	
3	Egress node measurement	N	N	Y	N	N	N	Y	N	
	Shared secret key in the 1st stage	—	—	1	—		—	0	—	

4	Egress node source basis	R	R	—	D	R	R	—	D	
	Egress node bit	1	0	—	0	0	1	—	1	
	Egress node polarization	V	H	—	45°	H	V	—	135°	
5	Ingress node detector basis	R	D	—	D	R	R	—	D	
6	Ingress node measurement	V	45°	—	45°	H	V	—	135°	
	Ingress node bit	1	0	—	0	0	1	—	1	

7	Ingress node reports basis	R	D	—	D	R	R	—	D	STAGE II
8	Egress node response	Y	N	—	Y	Y	Y	—	Y	
9	Shared secret key in the 1st stage	1	—	—	0	0	1	—	1	
10	The final shared secret key	1	—	1	0	0	1	0	1	

**Table 5:** Comparison of complexity order and efficiency of B92, BB84, and two-stage QKD protocols.

	B92	BB84	Two-stage QKD
Complexity order	2	4	2.86
Efficiency (%)	25	50	42.9

**Table 6:** Burst encryption time using RC4 and AES.

Burst size (packets/burst)	40000 packets	50000 packets	60000 packets	70000 packets	80000 packets
RC4 (ms)	**460.8**	**635.0**	**810.7**	**985.7**	**1160**
AES					
ECB (ms)	560.3	720.6	880.3	1035.5	1235.6
CBC (ms)	596.5	835.5	990.6	1185.6	1465.0
CFB (ms)	615.7	960.0	1185.7	1285.6	1507.3

The bold font refers to that the proposed algorithm produces better result than the existing algorithm.

**Table 7:** Burst decryption time using RC4 and AES.

Burst size (packets/burst)	40000 packets	50000 packets	60000 packets	70000 packets	80000 packets
RC4 (ms)	**410.7**	**527.0**	**700.6**	**867.3**	**973.3**
AES					
ECB (ms)	605.3	680.7	855.5	1276.3	1487.6
CBC (ms)	665.6	740.7	960.3	1327.6	1566.0
CFB (ms)	715.0	830.5	1010.6	1382.3	1545.7

The bold font refers to that the proposed algorithm produces better result than the existing algorithm.

**Table 8:** End-to-end delay calculation using RC4 and AES.

End-to-end delay time/burst size	40000 packets/burst	50000 packets/burst	60000 packets/burst	70000 packets/burst	80000 packets/burst
End-to-end delay time with RC4 based encryption (ms)	**1355.5**	**1766**	**2235**	**2697**	**3097**
End-to-end delay time with AES based encryption					
ECB (ms)	1650	2005	2460	3456	3687
CBC (ms)	1746	2180	2675	3357	3995
CFB (ms)	1815	2395	2920	3512	4017

The bold font refers to that the proposed algorithm produces better result than the existing algorithm.
